# Cohort Profile: The CArdiovascular Risk factors for hEalth Services research (CARhES) cohort study

**DOI:** 10.1093/ije/dyae015

**Published:** 2024-02-20

**Authors:** Isabel Aguilar-Palacio, MªJosé Rabanaque, Sara Castel, Lina Maldonado, Juan González-García, Luisa Compés, Sara Malo

**Affiliations:** Department of Preventive Medicine and Public Health, University of Zaragoza, Zaragoza, Spain; Grupo de Investigación en Servicios Sanitarios de Aragón (GRISSA), Fundación Instituto de Investigación Sanitaria de Aragón (IIS Aragón), Zaragoza, Spain; Network for Research on Chronicity, Primary Care, and Health Promotion (RICAPPS), ISCIII, Madrid, Spain; Department of Preventive Medicine and Public Health, University of Zaragoza, Zaragoza, Spain; Grupo de Investigación en Servicios Sanitarios de Aragón (GRISSA), Fundación Instituto de Investigación Sanitaria de Aragón (IIS Aragón), Zaragoza, Spain; Network for Research on Chronicity, Primary Care, and Health Promotion (RICAPPS), ISCIII, Madrid, Spain; Department of Preventive Medicine and Public Health, University of Zaragoza, Zaragoza, Spain; Grupo de Investigación en Servicios Sanitarios de Aragón (GRISSA), Fundación Instituto de Investigación Sanitaria de Aragón (IIS Aragón), Zaragoza, Spain; Grupo de Investigación en Servicios Sanitarios de Aragón (GRISSA), Fundación Instituto de Investigación Sanitaria de Aragón (IIS Aragón), Zaragoza, Spain; Department of Applied Economics, University of Zaragoza, Zaragoza, Spain; Network for Research on Chronicity, Primary Care, and Health Promotion (RICAPPS), ISCIII, Madrid, Spain; Biocomputing Unit, Aragón Health Sciences Institute (IACS), Zaragoza, Spain; Data Science for Health Services and Policy Research Group, Aragón Health Sciences Institute (IACS), Zaragoza, Spain; Grupo de Investigación en Servicios Sanitarios de Aragón (GRISSA), Fundación Instituto de Investigación Sanitaria de Aragón (IIS Aragón), Zaragoza, Spain; Departamento de Sanidad de Aragón, Dirección General de Asistencia Sanitaria, Zaragoza, Spain; Department of Preventive Medicine and Public Health, University of Zaragoza, Zaragoza, Spain; Grupo de Investigación en Servicios Sanitarios de Aragón (GRISSA), Fundación Instituto de Investigación Sanitaria de Aragón (IIS Aragón), Zaragoza, Spain; Network for Research on Chronicity, Primary Care, and Health Promotion (RICAPPS), ISCIII, Madrid, Spain

**Keywords:** Cardiovascular risk factors, cohort, health services, Real-World Data, healthcare inequalities

Key Features

- The CARhES (CArdiovascular Risk factors for hEalth Services research) study is a population cohort that uses Real-World Data to study the impact of health services and drug utilization on health outcomes in patients with cardiovascular risk factors and to identify potential healthcare inequalities. The ultimate goal is to develop strategies that enhance the medical management of these patients by promoting effective and equitable care.
- This is a population dynamic open cohort that includes all people aged ≥16 years old registered as users of the Aragón public health system (Spain) with hypertension, diabetes mellitus and/or dyslipidaemia from 2017 (number of participants: 446 998).
- Follow-up will last at least until the end of 2026, with annual waves of data extraction.
- Data on socio-demographics, clinical conditions, medications and use of health services are collected from clinical and administrative electronic databases.
- Due to its sensitive nature, the CARhES cohort data are not publicly accessible. However, researchers who wish to perform specific analyses, explore new methodologies or conduct cross-national comparisons related to healthcare utilization in patients with cardiovascular risk factors are encouraged to contact the Principal Investigators of the study: Isabel Aguilar-Palacio (iaguilar@unizar.es) and Sara Malo (smalo@unizar.es).

## Why was the cohort set up?

Cardiovascular disease (CVD) has a major individual, health and social impact. It is the leading cause of death worldwide, causes high disability, reduces the quality of life of those affected^1^ and has a major economic impact.^2^ The high frequency of its main individual risk factors, such as smoking, dyslipidaemia, high body mass index, hyperglycemia and high blood pressure, together with the marked ageing of the population, suggests that the frequency and impact of CVD are expected to remain unchanged in the coming decades,^3^ with an increase in certain vulnerable groups.^4^^,^^5^

CVD prevention, at both the population and individual levels, is a priority for current healthcare systems. Better control of risk factors for CVD is associated with a reduction in CVD risk and, consequently, with fewer hospital admissions and cardiovascular events.^6^ The most recent cardiovascular prevention guidelines^7^ stress the importance of adequate adherence to both lifestyle and pharmacological measures for effective CVD prevention. The guidelines emphasize the need to take into account patient preferences and individual needs when defining treatment goals and designing interventions.

Despite the existence of clinical guidelines, there is significant variability in patient care, e.g. the available diagnostic techniques,^8^ the different levels of care^9^ or their variable adherence to pharmacological treatments.^10^ The observed inequalities depend on the individual characteristics of the patients, the caregiving professionals involved and the health system itself.^11^^,^^12^ Additionally, factors such as the area in which a person lives, his or her socio-economic level, the social and family environment, age or gender may also contribute to defining population groups that show a specific use of health resources and, consequently, better or worse health outcomes.^13–15^

The Research Group in Health Services in Aragón (GRISSA) started in 2013 analysing data from the Aragón Workers' Health Study (AWHS).^16^ The AWHS cohort data were combined with Real-World Data, specifically routinely collected health data from the Aragón Health Service, such as electronic pharmacy records. The objective was to analyse the use of health services and cardiovascular preventive treatments.^17^^,^^18^ The research team gained significant expertise in the integration, analysis and interpretation of results obtained from large health databases. However, certain aspects, such as the aforementioned inequalities in healthcare utilization, could not be addressed due to the clinical-focused nature of the study. This highlighted the importance of analysing data at the population level.

To overcome the limitation, the CARhES cohort (CArdiovascular Risk factors for hEalth Services research) was set up in 2020. Its aim is to analyse the use of healthcare services and drugs in patients with risk factors for CVD residing in Aragón (Spain), in order to know the impact of healthcare utilization on health outcomes and to detect inequalities. The final objective is to develop strategies to improve the clinical management of these patients through effective and equitable care.

The CARhES study is a Real-World Data population-based cohort of secondary data from health services. Real-World Data, defined as data that are not collected in the setting of a randomized control trial, are a trustworthy resource for clinical and public health research.^19^ Particularly, the secondary use of routinely collected health data for research purposes is becoming more and more accessible and popular, as shown by the recent regulation of the European Health Data Space.^20^ It provides a unique information stream to describe the frequency and epidemiology of chronic diseases and its clinical practice. These sources may include linked data related to socio-demographic, geographical, clinical and therapeutic information, covering long time periods, offering an acceptable recording quality in most cases.

## Who is in the cohort?

The CARhES cohort is a population dynamic open cohort that uses Real-World Data and, more precisely, it makes secondary use of clinical and administrative data regularly collected in the Aragón Health Service. The inclusion criteria for the cohort are the following: the population aged ≥16 years old registered as users of the Aragón Health System with a medical diagnosis of hypertension (HTA), diabetes mellitus (DM) and/or dyslipidaemia (DL) and/or a prescription of at least one antidiabetic or lipid-lowering drug. In the case of HTA, only medical diagnosis is taken into account, given the widespread use of antihypertensive drugs for numerous pathologies.

Aragón is an Autonomous Community located in the north-east of Spain. It has a population of 1.3 million inhabitants, half of the population lives in the city of Zaragoza^21^ and it has a high level of ageing. The Aragón Health System, which covers >98% of the population, is structured in 8 health sectors organized into 123 basic healthcare areas, each of them served by a primary care centre and with populations of between 2000 and 5000 inhabitants.^22^ A comparison between the cohort participants and the Aragón population is available in Supplementary Table S1 (available as Supplementary data at *IJE* online).

**Figure 1.**
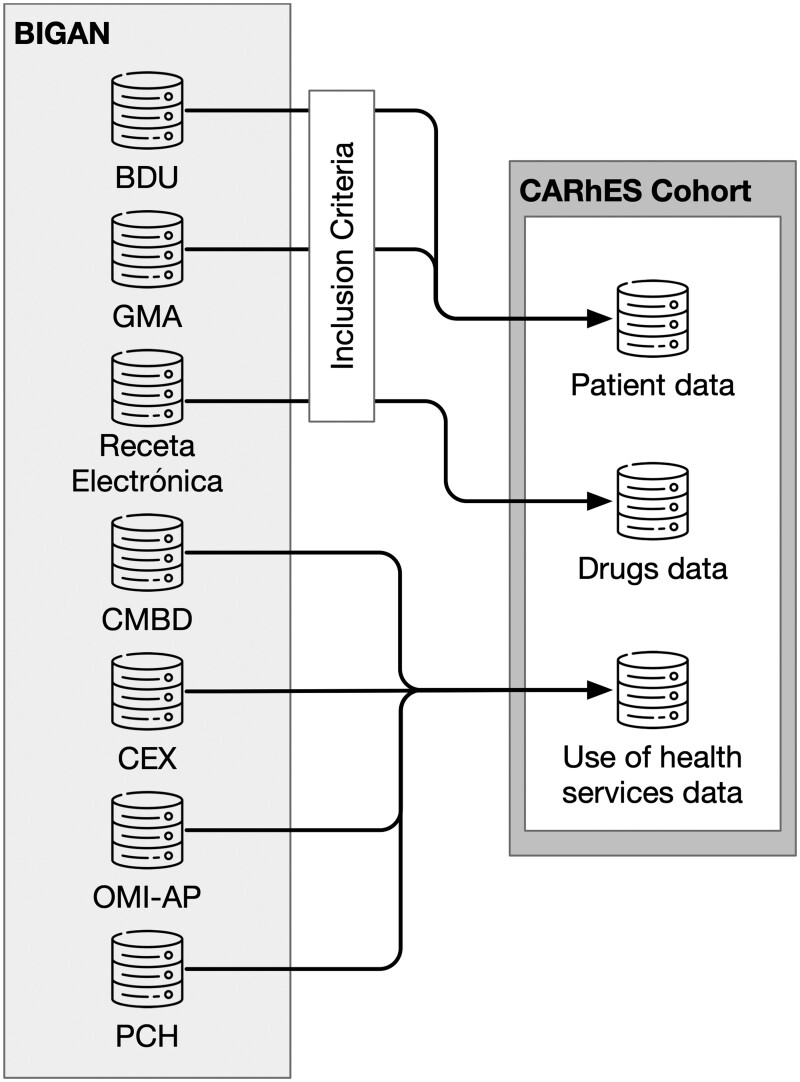
The CArdiovascular Risk factors for hEalth Services research (CARhES) cohort construction. BIGAN: a platform that integrates data from the Aragón Health Service (SALUD) information systems for its secondary use for research and policy-making; BDU: users database; GMA: adjusted morbidity groups; CMBD: hospital discharge records; CEX: specialist care; OMI-AP: primary care; PCH: emergency care; Receta Electrónica: electronic pharmacy records

Figure 1 presents the schema of the CARhES cohort construction. The data are extracted from BIGAN—a platform that integrates data from the Aragón Health Service (SALUD) information systems for their secondary use for research and policy-making (regulated by Law ORDEN SAN/1355/2018). This law provides the legal basis for secondary use of patients’ data, waiving the requirement of an active consent. The identification of patients who meet the inclusion criteria is carried out using three different data sources: BDU (Users Database), which provides information on age and coverage status to the Aragón public health system of all of the Aragonese population; GMA (Adjusted Morbidity Groups) database,^23^ which is used to detect diagnoses of interest considering the medical diagnoses available in primary care, hospital emergencies and hospital discharge records; and finally Receta Electrónica (electronic pharmacy records), which is used to identify pharmacological treatments. We identify specifically Anatomical Therapeutic Chemical (ATC) codes A10 (drugs used in diabetes), C02 (antihypertensives), C03 (diuretics), C07 (beta-blocking agents), C08 (calcium-channel blockers), C09 (agents acting on the renin–angiotensin system) and C10 (lipid-modifying agents). All data are pseudonymized in origin in the BIGAN platform with a unique code to allow its identification across the different linked databases.

After these data are provided by BIGAN, the research group proceeds to implement a more precise selection of patients included in the cohort, in order to get a detailed control of inclusion criteria. In the case of DM, we exclude women ≤50 years old with metformin prescription but no diagnosis of DM, as this drug can also be used for polycystic ovary syndrome. Subsequent classification of DM into type 1 or 2 is made according to the available primary care diagnoses [International Classification of Primary Care (ICPC) codes T89–T90 and literal labels]. In those people in whom it has not been clearly identified whether it is type 1 or 2 DM, drugs prescribed to these patients are reviewed, considering as type 2 DM those who only take oral antidiabetic drugs (ATC code A10B).

## How often have they been followed up?

The current ethics committee permit allows patient follow-up for 10 years, dating from 2017 to 2026. It is expected that this follow-up will be extended in the future with an annual update. For its management, there is a regular yearly extraction from the BIGAN databases and its integration in the CARhES cohort. There have been five waves of data consolidation extractions at the moment of writing this paper (from 1 January 2017 to 31 December 2021). In these 5 years of follow-up, there were 56 959 losses; 86.8% were due to the death of patients, 10.2% to the mobility of patients to other autonomous communities, 2.4% to health card expiry and 0.4% to loss of the right.

## What has been measured?

As can be observed in Figure 1, beyond the data used for the inclusion criteria, BIGAN provides information from four other data sources that are later integrated into the three CARhES cohort databases: patient data (both socio-demographic and clinical), drugs data and use of health services data. In Table 1, there is a detailed description of the information from the different CARhES databases.

**Table 1.** Data sources and original variables available in the CArdiovascular Risk factors for hEalth Services research (CARhES) cohort

Database and data source	Variables
Patient data	
BDU (users database)	Patient ID; date of birth; sex; registration date; administrative health area; living in an institution; economic activity; income band; pharmaceutical copayment; withdrawal; cause of withdrawal
GMA (adjusted morbidity groups)	Diagnosed chronic morbidity (yes/no): diabetes mellitus, heart failure, chronic obstructive pulmonary disease, hypertension, depression, HIV, ischaemic heart disease, stroke, chronic kidney disease, cirrhosis, osteoporosis, arthrosis, arthritis, dementia; number of chronic diseases; number of systems affected; morbidity burden; other diseases obtained from free text: dyslipidaemia and obesity
Drugs data	
Receta Electrónica (electronic pharmacy records)	Information obtained for each drug prescribed (electronic prescribing system) and received (dispensation database) by the patient: healthcare level of prescription (primary care or specialized care); prescription date; dispensation date; billing date; starting date; planned end date; ATC code; number of dispensed packages; defined daily doses; pharmaceutical form; dosage; price
Use of health services data	
CMBD (hospital discharge records)	Information obtained for each hospitalization: date of admission; re-entry; principal diagnosis^a^; secondary diagnoses^a^; surgical and diagnostic procedures; hospital; service of discharge; date of discharge; cause of discharge
PCH (emergency care)	Information obtained for each visit: date of visit; hospital; reason for visit; triage level; diagnosis; date of discharge; type of discharge
CEX (specialist care)	Information obtained for each visit: hospital; date of visit; medical specialty; origin
OMI-AP (primary care)	Information obtained for each visit: date of visit; type of visit; specialty; diagnosis (ICPC code and description); date of referral and specialty; family history; clinical parameters (date and value); diagnostic tests (date and type) including blood samples; lifestyle (physical activity, alcohol consumption, tobacco consumption, diet); anthropometric measures (body weight, height, abdominal perimeter, pulse rate, peripheral pulse)

ATC, Anatomical, Therapeutic, Chemical classification system; ICPC, International Classification of Primary Care; ID, identification.

a Using the International Classification of Diseases, 10th edition (ICD-10).

Every time a patient contacts the healthcare system, a record is generated containing different types of information that is captured in BIGAN. Once a year, there is an extraction of the data from the BIGAN platform that is provided to the research group. It is at this point that the CARhES cohort integration takes place, together with the cleansing quality review of the information and the fine-grain inclusion patient selection. In addition, we compute a number of new variables that are useful for facilitating further analyses. These variables fall into the following categories: patient profiling, health services and drugs use, inequalities data and health outcomes data.

The patient profiles for each risk factor for CVD are computed using sex, age group and morbidity burden, and serve as a proxy for the complexity of the patients. Profiles are created using a two-step cluster analysis approach on the mentioned variables and the optimal number of clusters was determined using the Bayesian Information Criterion. We obtained seven different profiles for patients with type 2 DM, seven for patients with HTA and eight for patients with DL.

Health services use computed variables that include the number of medical visits to primary care or to a specialist doctor for a certain period of time and the proportion of patients who are prescribed a particular therapeutic subgroup, among others. The study of adherence to medication is analysed according to the different phases defined by The Ascertaining Barriers to Compliance (ABC) taxonomy^24^ and conceptualized based on the Medication Adherence Reporting Guideline (EMERGE)^25^ and the TEOS (Timelines–Events–Objectives–Sources) framework,^26^ in order to improve its transparency and comparability.

Gender, place of residence and socio-economic level are included for each individual to analyse inequalities. Regarding the socio-economic level, pharmacy copayment levels and the type of activity (employed, unemployed, pensioner) are combined to provide a good approximation for the socio-economic position of the individual.^27^ Deprivation of the basic healthcare area of residence is considered using a deprivation index.^28^ Areas of residence are also categorized into rural or urban according to the classification followed by the Aragón government.^29^

Data on major adverse cardiovascular events (myocardial infarction and stroke), as well as other health outcomes, such as mortality or the occurrence of adverse effects, were obtained. Major adverse cardiovascular event diagnoses correspond to codes I21 and I60–I63 of the International Classification of Diseases (ICD-10). Major adverse cardiovascular events are obtained from hospital discharge records (CMBD) (hospital discharge records) and emergency care (PCH) (see Figure 1). Mortality is identified from BDU (users database) and the cause of mortality can be obtained in those patients with a hospitalization or an emergency admission.

## What has it found?

**Figure 2.**
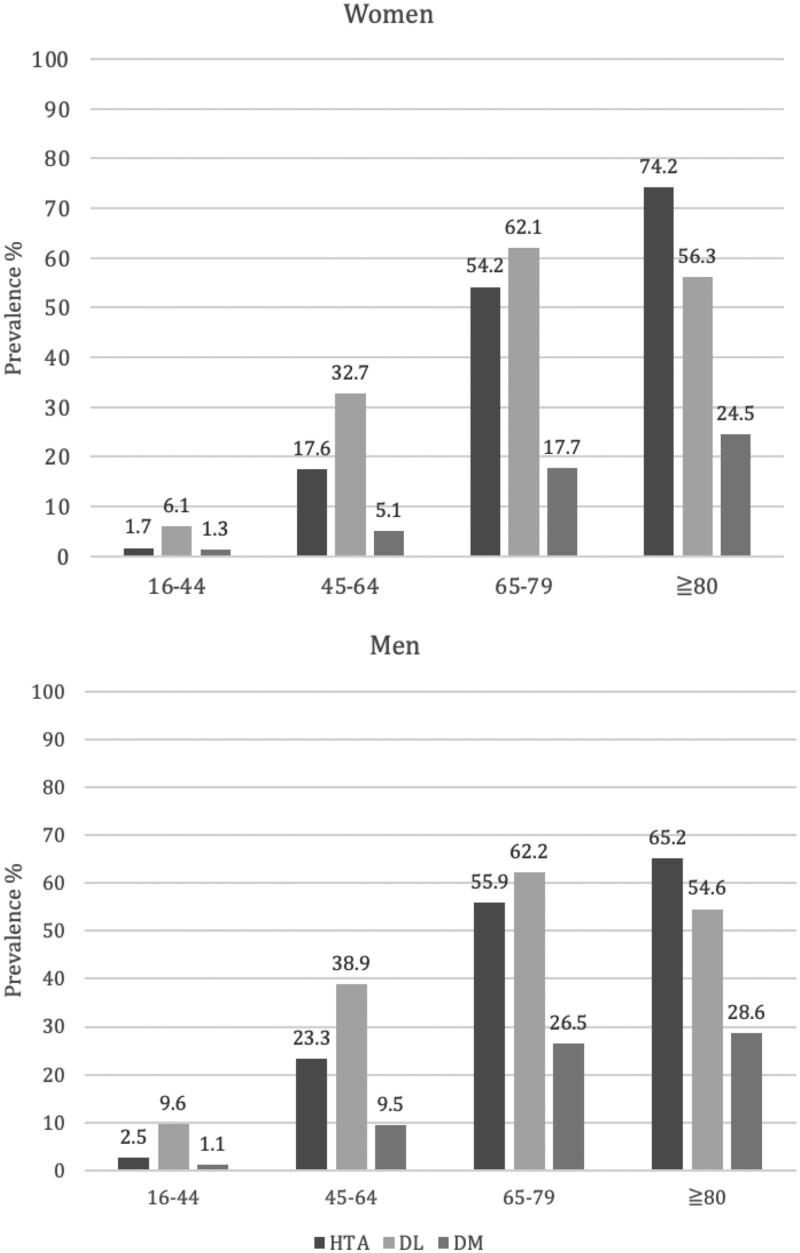
Prevalence of cardiovascular risk factors in the CArdiovascular Risk factors for hEalth Services research (CARhES) cohort by sex and age groups, 2017. HTA, hypertension; DL, dyslipidaemia; DM, diabetes mellitus. Population of reference: all the Aragón Health System users for each sex and group of age

In 2017, the CARhES cohort started with the inclusion of 446 998 individuals. From these individuals, 252 508 fulfil HTA criteria, 332 644 fulfil DL criteria and 96 709 fulfil DM criteria. The majority of individuals (57.8%) had only one risk factor for CVD and 10.4% of the patients included in the CARhES cohort had three of them. Information about the prevalence of risk factors for CVD in the CARhES cohort by sex and groups of age at the beginning of the follow-up can be found in Figure 2.

In the 2021 update, the number of patients in the CARhES cohort increased to 532 568. It represents an incidence of 11.1 cases of HTA per 1000 people per year, 20.3 cases of DL and 8.9 cases of DM. The geographical distribution of risk factors for CVD showed differences for the three of them analysed (Supplementary Figure S1, available as Supplementary data at *IJE* online).

In Table 2, the baseline characteristics of the CARhES cohort are available by risk factor for CVD. There are similar proportions of men and women, and the age group of 45–64 years has the highest number of individuals. People have low socio-economic status and live mainly in urban areas. They have a high morbidity burden, based on the number and severity of diagnosed diseases.^23^

**Table 2.** The CArdiovascular Risk factors for hEalth Services research (CARhES) cohort socio-demographic and clinical baseline characteristics in 2017 by cardiovascular risk factor

Characteristic	Total [*n* (%)]	Hypertension [*n* (%)]	Dyslipidaemia [*n* (%)]	Diabetes mellitus [*n* (%)]
Sex				
Men	220 618 (49.4)	119 812 (47.4)	166 512 (50.1)	52 867 (54.7)
Women	226 380 (50.6)	132 696 (52.6)	166 132 (49.9)	43 842 (45.3)
Age (years)				
16–44	48 844 (10.9)	10 521 (4.2)	38 021 (11.4)	5654 (5.9)
45–64	172 654 (38.6)	78 176 (30.9)	134 904 (40.6)	28 068 (29.0)
65–79	139 173 (31.2)	94 949 (37.6)	105 897 (31.8)	37 711 (39.0)
≥80	86 327 (19.3)	68 862 (27.3)	53 822 (16.2)	25 276 (26.1)
Socio-economic level				
Employed <18K^a^	83 294 (18.6)	30 326 (12.0)	64 811 (19.5)	11 087 (11.5)
Employed ≥18K^a^	59 798 (13.4)	22 589 (8.9)	47 118 (14.2)	6899 (7.1)
Unemployed	22 810 (5.1)	9726 (3.9)	17 419 (5.2)	4125 (4.3)
Mutualist^b^	3371 (0.8)	1764 (0.70)	2324 (0.7)	594 (0.6)
Pensioner <18K^a^ and free medicines	190 113 (42.5)	135 234 (53.6)	134 679 (40.5)	54 027 (55.9)
Pensioner ≥18K^a^	72 666 (16.3)	46 474 (18.4)	55 454 (16.6)	17 346 (17.9)
Other	14 946 (3.3)	6395 (2.5)	10 839 (3.26)	2631 (2.7)
Area of residence				
Rural	130 767 (29.3)	77 047 (30.5)	94 919 (828.5)	29 359 (30.4)
Urban	316 230 (70.7)	175 460 (69.5)	237 724 (71.5)	67 350 (69.6)
Morbidity burden^c^ [mean (SD)]	7.1 (4.8)	8.5 (5.0)	7.1 (4.8)	9.5 (5.5)
Comorbidities				
Obesity	67 368 (15.1)	49 826 (19.7)	47 937 (14.4)	21 547 (22.3)
Heart failure	15 207 (3.4)	11 843 (4.7)	10 384 (3.1)	6111 (6.3)
Ischaemic heart disease	30 066 (6.8)	19 871 (7.9)	27 033 (8.1)	10 548 (10.9)
Stroke	16 656 (3.7)	12 436 (4.9)	13 117 (3.9)	5543 (5.7)
COPD	25 205 (5.7)	17 444 (6.9)	18 562 (5.6)	7585 (7.8)
Chronic kidney disease	42 458 (9.5)	39 351 (15.6)	29 766 (8.9)	14 111 (14.6)
Cirrhosis	11 623 (2.6)	7344 (2.9)	8801 (2.6)	4127 (4.3)
Osteoporosis	50 140 (11.2)	33 203 (13.1)	37 812 (11.4)	8484 (8.8)
Arthritis	11 146 (2.5)	6791 (2.7)	8348 (2.5)	2227 (2.3)
Dementia	12 203 (2.7)	9392 (3.7)	7848 (2.4)	3619 (3.7)
Depression	67 109 (15.0)	41 247 (16.3)	51 474 (15.5)	15 377 (15.9)
Total	446 998 (100)	252 508 (56.5)	332 644 (74.4)	96 709 (21.6)

COPD, chronic obstructive pulmonary disease.

a €18 000 per year.

b Civil servant.

c Summary index of the clinical diagnosed conditions.

Patients with risk factors for CVD have a high use of healthcare services and drugs. Table 3 provides a comprehensive overview of patients included in 2017 in the CARhES cohort and their healthcare and drug utilization within 1 year of inclusion. Nearly all patients visited their primary care physician, averaging ∼20 visits per year, and approximately two-thirds of these patients sought care from specialist physicians. Pharmacological burden ranged from 6.8 different therapeutical subgroups in 1 year in patients with DL to 8.2 in patients with DM. The proportion of individuals receiving specific treatment was the highest among those with HTA (88.8%). Those with DM exhibited the highest utilization of healthcare services and had the greatest likelihood of hospital admissions and visits to the emergency department. Lastly, their drug use also exceeded those of patients with other risk factors for CVD.

**Table 3.** The CArdiovascular Risk factors for hEalth Services research (CARhES) cohort use of health services and drugs in the first year of follow-up by cardiovascular risk factor

Use of health services and drugs	Hypertension (*n* = 252 508)	Dyslipidaemia (*n* = 332 644)	Diabetes mellitus (*n* = 96 709)
People with at least one visit to primary care doctor [*n* (%)]	246 083 (97.5)	319 776 (96.1)	94 531 (97.7)
People with at least one visit to primary care nurse [*n* (%)]	207 968 (82.4)	250 317 (75.3)	85 138 (88.0)
Number of visits to primary care^a^			
Mean (SD)	19.8 (18.0)	18.8 (18.0)	23.9 (20.8)
Median (P25–P75)	13 (8–22)	12 (7–21)	16 (10–26)
People with at least one visit to specialist doctor [*n* (%)]	172 671 (68.4)	222 284 (66.8)	70 963 (73.4)
Number of visits to specialist doctor^a^			
Mean (SD)	6.8 (7.6)	6.9 (7.9)	7.7 (8.9)
Median (P25–P75)	4 (2–7)	4 (2–8)	4 (2–8)
People with at least one visit to specialist doctor [*n* (%)]			
Cardiologist	35 892 (14.2)	44 197 (13.3)	15 880 (16.4)
Endocrinologist	18 271 (7.2)	24 250 (7.3)	16 518 (17.1)
Vascular surgery	6899 (2.7)	8728 (2.6)	3500 (3.6)
Ophthalmologist	50 005 (19.8)	57 491 (17.3)	23 233 (24.0)
Nephrologist	7545 (3.0)	7531 (2.3)	3662 (3.8)
People with at least one hospital admission [*n* (%)]	38 544 (15.3)	48 965 (14.7)	19 468 (20.1)
Number of hospital admissions^a^			
Mean (SD)	1.5 (1.1)	1.5 (1.1)	1.6 (1.2)
Median (P25–P75)	1 (1–2)	1 (1–2)	1 (1–2)
People with at least one visit to emergency service [*n* (%)]	74 705 (29.6)	98 502 (29.6)	32 782 (33.9)
Number of emergency service visits^a^			
Mean (SD)	1.8 (1.5)	1.8 (1.6)	1.9 (1.7)
Median (P25–P75)	1 (1–2)	1 (1–2)	1 (1–2)
People with at least one drug prescription [*n* (%)]			
Antihypertensives (ATC codes: C02, C03, C07–C09)	224 129 (88.8)	170 322 (51.2)	68 762 (71.1)
Lipid-lowering drugs (ATC code: C10)	117 360 (46.5)	216 066 (65.0)	59 092 (61.1)
Anti-diabetics (ATC code: A10)	54 093 (21.4)	61 973 (18.6)	84 001 (86.9)
Number of prescriptions among treated^b^ [mean (SD)]			
Antihypertensives (ATC codes: C02, C03, C07–C09)	23.8 (14.6)	27.5 (17.5)	30.1 (18.6)
Lipid-lowering drugs (ATC code: C10)	13.0 (4.8)	12.3 (4.5)	13.9 (5.7)
Anti-diabetics (ATC code: A10)	21.2 (12.0)	22.0 (12.5)	19.7 (11.0)
Pharmacological burden (all ATC codes),^c^ mean (SD)	7.3 (3.8)	6.8 (3.9)	8.2 (4.1)

ATC, Anatomical Therapeutic Chemical code.

a Mean and SD calculated in those with at least one visit.

b Number of prescriptions refers to number of dispensations.

c Pharmacological burden was estimated based on the number of different pharmacological subgroups dispensed.

When we analysed the use of healthcare services by the profiles obtained, greater complexity was associated with greater use of health services. In the case of primary care utilization in patients with DM, the least complex patients’ profiles had an average of 7.6 visits per year to their primary care physician, whereas the most complex had an average of 12.3 visits per year.

We identified healthcare inequalities for the different patient profiles by socio-economic level and area of residence. People living in depopulated areas had a higher attendance to primary healthcare than those living in very populated areas. In relation to socio-economic levels, pensioners and employed people earning <€18 000 per year showed the highest attendance to primary healthcare in comparison with people with high incomes, as has been described using other sources of information.^30^

The risk of suffering a major adverse cardiovascular event by sex and the influence of different factors on it have been analysed using different machine-learning techniques. Our findings reveal that the most important contributor for predicting CVD risk was age followed by adherence to antidiabetic medication. The importance of the rest of the variables varied across different models, but DM was the most important risk factor for CVD in most models developed for women.

Lastly, it is crucial to recognize the impact of the COVID-19 pandemic on the management and behaviour of individuals with risk factors for CVD. We conducted a study^31^ to compare adherence patterns before and during the COVID-19 pandemic among individuals who were first prescribed statins for primary CVD prevention. The study findings revealed the presence of four distinct trajectories of statin adherence across the pandemic. Older age, being a pensioner and having a higher morbidity burden were associated with higher and consistent adherence over time. Conversely, younger individuals, those employed with an annual income of <€18 000 or unemployed and individuals without comorbidities were more likely to fall into the poor adherence trajectory.

## What are the main strengths and weaknesses?

The main strength is that it is a population-based cohort that includes all patients with a risk factor for CVD in an ageing population in southern Europe with a health system based on the principles of universal, equitable, free access and fairness of financing.^32^ Therefore, the results obtained are generalizable to other populations with similar settings, which are common in European countries. Data obtained from BIGAN are subjected to rigorous quality control procedures during the whole data lifecycle. These procedures aim to enhance the reliability, validity, quality and suitability of the CARhES cohort data for research purposes from its origin. Clinical conditions of the patients of the cohort are obtained from GMA—a source of information that combines diagnoses from CMBD, PCH and OMI-AP (primary care), which makes this a high-sensitivity classification^23^ and whose validity has been amply demonstrated.^33^^,^^34^ Also, by combining clinical diagnoses with drug consumption, highly sensitive inclusion criteria can be established to effectively identify individuals with risk factors for CVD. Finally, the good performance of the cohort in terms of findings and the low maintenance cost, due to its secondary-use nature, are expected to lead a larger follow-up.

The cohort also has some limitations worth mentioning. The use of administrative and electronic health databases is limited by the quality of the data recorded, as they have not been designed for research. Therefore, some data, especially those related to lifestyles, may not be fully collected or regularly updated. In any case, the quality of these databases has increased significantly in recent years and regular quality controls of the variables have been carried out by the CARhES cohort. Regarding private healthcare, the CARhES cohort includes information on hospital admissions in private hospitals, but does not include visits to primary care professionals, nor to specialist doctors in private healthcare. Even though we estimate minor use of private healthcare, this is an area for improvement and we hope to incorporate these data in the near future. The CARhES cohort is not linked to the Mortality Registry, so the cause of death is only available when it happens in a healthcare facility. Finally, due to factors related to patient identification, the smallest area of residence available is the basic healthcare area. Work is being done to improve a finer geo-localization of the patients while preserving individuals’ privacy.

## Can I get hold of the data? Where can I find out more?

Data of this cohort are not freely available due to sensitive information, but we are open to collaborations aligned with the main objectives of the cohort. Researchers interested in conducting data analyses, developing new methodological approaches or doing cross-national comparisons should contact the Principal Investigators (Isabel Aguilar-Palacio iaguilar@unizar.es and Sara Malo smalo@unizar.es).

## Ethics approval

The protocol of the CARhES cohort was approved by the Clinical Research Ethics Committee of Aragón (CEICA. PI21/148).

## Supplementary Material

dyae015_Supplementary_Data

## Data Availability

See ‘Can I get hold of the data?’ above.

## References

[1] World Health Organization. *Mortality and Global Health Estimates. Global Health Observatory*. World Health Organization. 2020. https://www.who.int/data/gho/data/themes/mortality-and-global-health-estimates (12 January 2024, date last accessed).

[2] Timmis A , Townsend N, Gale C et al; ESC Scientific Document Group. European Society of Cardiology: cardiovascular disease statistics 2017. Eur Heart J 2018;39:508–79. 29190377 10.1093/eurheartj/ehx628

[3] Armario P , Brotons C, Elosua R et al Statement of the Spanish Interdisciplinary Vascular Prevention Committee on the updated European Cardiovascular Prevention Guidelines. Clin Investig Arterioscler 2021;33:85–107. 10.1016/j.arteri.2020.11.004 33495044

[4] Haeberer M , León-Gómez I, Pérez-Gómez B, Tellez-Plaza M, Rodríguez-Artalejo F, Galán I. Social inequalities in cardiovascular mortality in Spain from an intersectional perspective. Rev Esp Cardiol 2020;73:282–89. 31784414 10.1016/j.rec.2019.07.022

[5] Pérez-Hernández B , García-Esquinas E, Graciani A et al Social inequalities in cardiovascular risk factors among older adults in Spain: the seniors-ENRICA study. Rev Española Cardiol (English Ed) 2017;70:145–54. 10.1016/j.rec.2016.05.010 27519455

[6] George MG , Tong X, Bowman BA. Prevalence of cardiovascular risk factors and strokes in younger adults. JAMA Neurol 2017;74:695–703. 28395017 10.1001/jamaneurol.2017.0020 PMC5559660

[7] Visseren FLJ , MacH F, Smulders YM et al; ESC Scientific Document Group. 2021 ESC Guidelines on cardiovascular disease prevention in clinical practice. Eur Heart J 2021;42:3227–337. 34458905 10.1093/eurheartj/ehab484

[8] Valcárcel-Nazco C , Alonso-Modino D, Montón-Álvarez F et al Variability in the use of neuroimaging techniques for diagnosis and follow-up of stroke patients. Neurologia (Engl Ed) 2019;34:360–66. 28431835 10.1016/j.nrl.2017.02.001

[9] Kirchberger I , Amann U, Heier M, Thilo C, Peters A, Meisinger C. Factors associated with emergency services use by patients with recurrent myocardial infarction. J Cardiovasc Nurs 2017;32:409–18. 27428355 10.1097/JCN.0000000000000359

[10] Menditto E , Cahir C, Aza-Pascual-Salcedo M et al Adherence to chronic medication in older populations: application of a common protocol among three european cohorts. Patient Prefer Adherence 2018;12:1–13. 30323567 10.2147/PPA.S164819 PMC6179242

[11] Barros P , Barry M, Brand H et al Expert Panel on effective ways of investing in Health (EXPH), Report of Disruptive Innovation. 2016. https://health.ec.europa.eu/system/files/2019-11/008_competition_healthcare_providers_en_0.pdf (12 January 2024, date last accessed).

[12] Kardas P , Lewek P, Matyjaszczyk M. Determinants of patient adherence: a review of systematic reviews. Front Pharmacol 2013;4:91. 23898295 10.3389/fphar.2013.00091 PMC3722478

[13] Hyun KK , Redfern J, Patel A et al Gender inequalities in cardiovascular risk factor assessment and management in primary healthcare. Heart 2017;103:492–98. 28249996 10.1136/heartjnl-2016-310216

[14] Sánchez-Recio R , Alonso JP, Aguilar-Palacio I. The use of health care services in the Great Recession: evaluating inequalities in the Spanish context. Gac Sanit 2020;34:245–52. 32005597 10.1016/j.gaceta.2019.10.009

[15] Asthana S , Moon G, Gibson A, Bailey T, Hewson P, Dibben C. Inequity in cardiovascular care in the English National Health Service (NHS): a scoping review of the literature. Health Soc Care Community 2018;26:259–72. 27747961 10.1111/hsc.12384

[16] Casasnovas JA , Alcaide V, Civeira F et al Aragon workers’ health study—design and cohort description. BMC Cardiovasc Disord 2012;12:45. 22712826 10.1186/1471-2261-12-45 PMC3439398

[17] Aguilar-Palacio I , Malo S, Feja C et al Risk factors control for primary prevention of cardiovascular disease in men: Evidence from the Aragon Workers Health Study (AWHS). PLoS One 2018;13:e0193541. 29474499 10.1371/journal.pone.0193541 PMC5825136

[18] Malo S , Aguilar-Palacio I, Feja C et al Different approaches to the assessment of adherence and persistence with cardiovascular-disease preventive medications. Curr Med Res Opin 2017;33:1329–36. 28422521 10.1080/03007995.2017.1321534

[19] Makady A , de Boer A, Hillege H, Klungel O, Goettsch W; (on behalf of GetReal Work Package 1). What is real-world data? A review of definitions based on literature and stakeholder interviews. Value Heal 2017;20:858–65. 10.1016/j.jval.2017.03.008 28712614

[20] European Commission. *Regulation of the European Parliament and of the Council on the European Health Data Space*. COM(2022)197. European Commission. 2021. https://ec.europa.eu/transparency/documents-register/detail?ref=C(2014)4995&lang=en%0Ahttps://ec.europa.eu/transparency/documents-register/detail?ref=SWD(2016)226&lang=en%0Ahttps://ec.europa.eu/transparency/documents-register/detail?ref=SWD(2021)150&lang= (12 January 2024, date last accessed).

[21] Gobierno de Aragón. *Instituto Aragonés de Estadística.* https://www.aragon.es/organismos/departamento-de-economia-planificacion-y-empleo/direccion-general-de-economia/instituto-aragones-de-estadistica-iaest (12 January 2024, date last accessed).

[22] Gobierno de Aragón. *Sectores Sanitarios*. Directorio de Centros de Asistencia. Gobierno de Aragón. https://www.aragon.es/sectores-sanitarios (12 January 2024, date last accessed).

[23] Monterde D , Vela E, Clèries M. Los grupos de morbilidad ajustados: nuevo agrupador de morbilidad poblacional de utilidad en el ámbito de la atención primaria. Atención Primaria 2016;48:674–82. 27495004 10.1016/j.aprim.2016.06.003 PMC6879171

[24] Vrijens B , De Geest S, Hughes DA et al; ABC Project Team. A new taxonomy for describing and defining adherence to medications. Br J Clin Pharmacol 2012;73:691–705. 22486599 10.1111/j.1365-2125.2012.04167.x PMC3403197

[25] De Geest S , Zullig LL, Dunbar-Jacob J et al ESPACOMP medication adherence reporting guideline (EMERGE). Ann Intern Med 2018;169:30–35. 29946690 10.7326/M18-0543 PMC7643841

[26] Dima AL , Allemann SS, Dunbar-Jacob J, Hughes DA, Vrijens B, Wilson IB. TEOS: a framework for constructing operational definitions of medication adherence based on Timelines–Events–Objectives–Sources. Br J Clin Pharmacol 2021;87:2521–33. 33220097 10.1111/bcp.14659

[27] Aguilar-Palacio I , Maldonado L, Malo S et al COVID-19 inequalities: individual and area socioeconomic factors (Aragón, Spain). IJERPH 2021;18:6607. 34205348 10.3390/ijerph18126607 PMC8296401

[28] Compés Dea ML , Olivan Bellido E, Feja Solana C et al Construcción de un índice de privación por zona básica de salud en Aragón a partir de datos de censo de 2011. Rev Esp Salud Publica 2018;92:e201812087. 30531710 PMC11587363

[29] Gobierno de Aragón. *Orden de 20 de febrero de 2015, del Consejero de Sanidad, Bienestar Social y Familia, por la que se actualiza la clasificación de las zonas de salud de la Comunidad Autonoma de Aragón a efectos de planificación farmaceutica*. BOA, 2015, num. 36. https://www.boa.aragon.es/cgi-bin/EBOA/BRSCGI?CMD=VEROBJ&MLKOB=839119044747 (12 January 2024, date last accessed).

[30] Aguilar-Palacio I , Carrera-Lasfuentes P, Solsona S, Sartolo MT, Rabanaque MJ. Health-care utilization in elderly (Spain 2006-2012): influence of health status and social class. Aten Primaria 2016;48:235–43. 26388467 10.1016/j.aprim.2015.01.016 PMC6877833

[31] Malo S , Maldonado L, Rabanaque MJ et al Patterns of statin adherence in primary cardiovascular disease prevention during the pandemic. Front Pharmacol 2022;13:980391. 36452233 10.3389/fphar.2022.980391 PMC9702325

[32] Bernal E , Sandra D, Juan G-A et al *Spain Health System Review*. Health Syst Transit. 2018. https://iris.who.int/bitstream/handle/10665/330195/HiT-20-2-2018-eng.pdf?isAllowed=y&sequence=11&utm_medium=email&utm_source=transaction (12 January 2024, data last accessed).

[33] Bernal JL , Barrabés JA, Íñiguez A et al Clinical and administrative data on the research of acute coronary syndrome in spain. minimum basic data set validity. Rev Esp Cardiol (Engl Ed) 2019;72:56–62. 29747944 10.1016/j.rec.2018.01.026

[34] Lopez-de-Andres A , Jiménez-García R, Aragón-Sánchez J et al National trends in incidence and outcomes in lower extremity amputations in people with and without diabetes in Spain, 2001-2012. Diabetes Res Clin Pract 2015;108:499–507. 25866357 10.1016/j.diabres.2015.01.010

