# Supplementary material for: Cohort Profile: The CArdiovascular Risk factors for hEalth Services research (CARhES) cohort study
Source: Int J Epidemiol. 2024 Feb 20;53(2):dyae015. doi: 10.1093/ije/dyae015 (PMC10879753; doi:10.1093/ije/dyae015)
Supplement: dyae015_Supplementary_Data [file dyae015_supplementary_data.pdf]

Supplementary material

Table S1. Sociodemographic comparison between Aragón population and the CArdiovascular Risk factors for hEalth Services research (CARhES) cohort population.

		Aragón population N(%)	CARhES population N(%)
Sex			
	Men	532959 (48.8)	220618 (49.4)
	Women	558258 (51.2)	226380 (50.6)
Age (years)			
	16-44	446299 (40.9)	48844 (10.9)
	45-64	370697 (34.0)	172654 (38.6)
	65-79	172943 (15.8)	139173 (31.2)
	≥80	101278 (9.28)	86327 (19.3)
Socioeconomic level			
	Employed <18K	411980 (37.8)	94849 (21.2)
	Employed ≥18K	224084 (20.5)	70426 (15.8)
	Mutualist	8475 (0.8)	3368 (0.8)
	Pensioner <18K and free medicines	267313 (24.5)	190346 (42.6)
	Pensioner ≥18K	95700 (8.8)	73060 (16.3)
	Other	83520 (7.7)	14945 (3.3)
Area of residence			
	Rural	303750 (27.8)	130767 (29.3)
	Urban	787467 (72.2)	316230 (70.7)

N: number; % percentage; 18K: 18,000 € per year; Mutualist: civil servants.

Figure S1. **Spatial distribution of Cardiovascular Risk Factors in Aragón by sex and basic healthcare area. Results: standardized prevalence in% (Direct standardization by age).**

Women

1 2 3

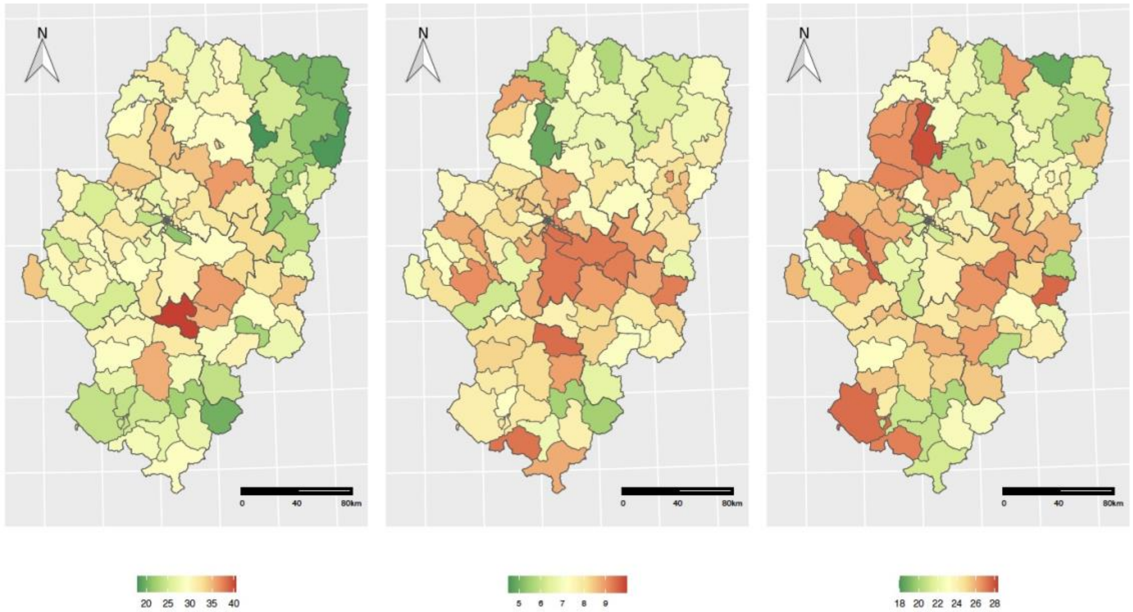

Men

1 2 3

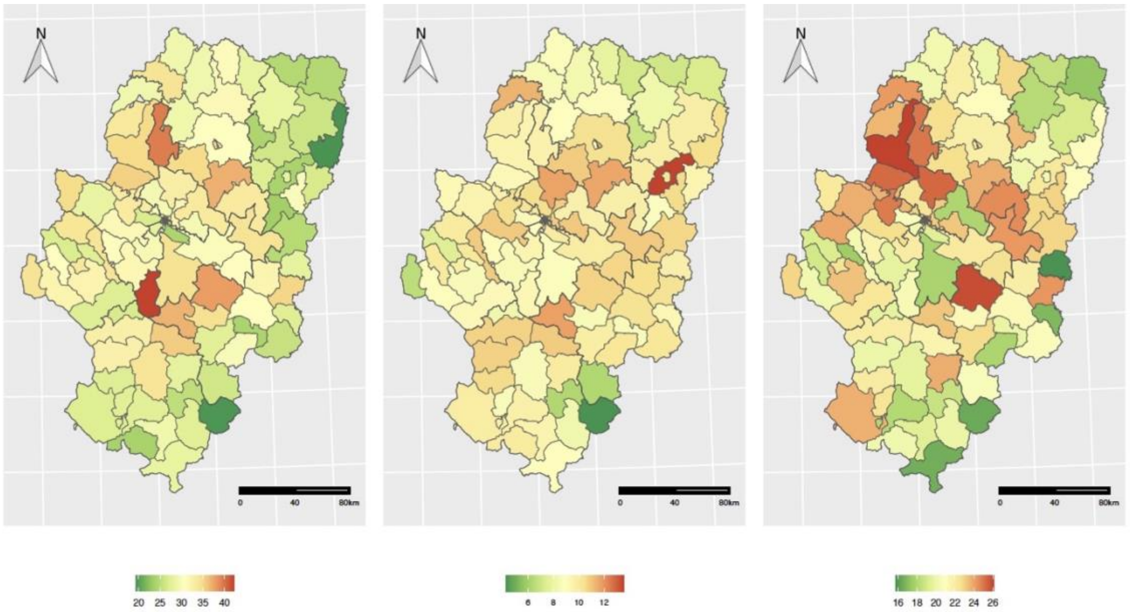

1. Dyslipidaemia; 2. Diabetes mellitus; 3. Hypertension
